# Particularities of suicide attempts in late adolescence in Tunisia

**DOI:** 10.1192/j.eurpsy.2022.704

**Published:** 2022-09-01

**Authors:** R. Boukhchina, A. Aissa, I. Kammoun, Y. Zgueb, S. Madouri, U. Ouali, R. Jomli

**Affiliations:** 1 Razi hospital, Psychiatry A Department, Manouba, Tunisia; 2 Razi hospital, Psychiatry A Department, manouba, Tunisia

**Keywords:** risk factors, suicide attempts, late adolescence

## Abstract

**Introduction:**

Suicide attempts in late adolescence deserves special attention.

Identifying particularities of suicidal behavior in this age group seems important in order to detect suicidal ideations.

**Objectives:**

Describe the characteristics of suicide attempts in late adolescence among hospitalized patients.

**Methods:**

This is a retrospective descriptive study that was conducted in our psychiatry department in Razi Hospital, Tunisia. It focused on a population of Tunisian adolescents aged between 15 and 19 years old and who were hospitalized after a suicide attempt between January, 1st 2010 and November,15th 2018.We used a pre-established questionnaire that explored the sociodemographic and clinical data of patients.

**Results:**

Thirty adolescents were included. Twenty-three of them (77%) were female. Mean age of suicidal adolescents was 16.5 years. They were mostly living with their families (80%). Intentional drug ingestion was reported in 56% of cases. Half of the adolescents were indifferent regard the suicide attempt. Conflictual family environment was reported to be a triggering factor of the suicidal thoughts in 60% of cases, and romantic breakup in 20% of cases. In fact, these adolescents were diagnosed with adjustment disorder with depressed mood in 47% of them and depression (28%).Adolescent suicide attempts were correlated with a conflictual family environment (p=0.04) and the presence of academic difficulties (p<10-3).

**Conclusions:**

Family dysfunction and conflictual environment are predictors of suicide risk in the late adolescence. Prevention strategies should be reviewed and focus more on these factors.

**Disclosure:**

No significant relationships.

